# The knowledge, attitudes and practices of parents of children with asthma in 29 cities of China: a multi-center study

**DOI:** 10.1186/1471-2431-13-20

**Published:** 2013-02-04

**Authors:** Jing Zhao, Kunling Shen, Li Xiang, Guoqing Zhang, Meng Xie, Juan Bai, Qiyi Chen

**Affiliations:** 1 Capital Institute of Pediatrics, Beijing, China; 2 Beijing Children’s Hospital, Beijing, China

**Keywords:** Asthma, Knowledge, Attitudes, Practices, Parents, China

## Abstract

**Background:**

Asthma is becoming increasingly prevalent among children in China. Poor parent knowledge and attitudes often contribute to inappropriate management practices, leading to deficiencies in the care process. We aimed to document the knowledge, attitudes and practices (KAP) of parents of children with asthma and analyze how knowledge and attitudes relate to practices. Our secondary objective was to identify the factors associated with parent KAP scores.

**Methods:**

A KAP questionnaire was distributed to parents caring for 2960 children (0–14 years) diagnosed with asthma for at least 3 months from China’s 29 provinces. A 50-item questionnaire was devised for this cross-sectional survey based on a comprehensive review of the subject. Questionnaires were scored on 30 items regarding parent asthma-related KAP, with one point for every correct response and a possible range of 0–13 for knowledge, 0–7 for attitudes and 0–10 for practices. Higher scores indicated better KAP. Chi-squared tests and logistic regression were used to identify factors associated with practices and combined KAP scores.

**Results:**

The response rate was 83.95% (2485/2960). Only 18.31% (455/2485) of parents correctly answered ≥ 60% of the knowledge questions (mean = 5.69). Most (89.85%; 2226/2485) gave positive responses to ≥ 60% of the attitude questions (mean = 5.23) while 67.89% (1687/2485) correctly answered ≥ 60% of the practices questions (mean = 6.19). Knowledge and attitudes were positively associated with pulmonary function testing, regular physician visits, monitoring with a peak flow meter and the Children’s Asthma Control Test questionnaire, avoidance of asthma triggers, using an inhaled β2 receptor agonist and adherence to medication regimen (*p* ≤ 0.05). Attitudes were also associated with allergen testing. In logistic regression analysis, high KAP scores (dichotomized by a cut-off score of 18) were positively associated with food allergy, rhinitis, physician visits, frequency of visits and parent education (*p* < 0.05, OR > 1).

**Conclusions:**

Generally, the parents’ KAP were poor. A gap between recommended and actual practice was observed, which may be related to inadequate knowledge about and poor attitudes toward childhood asthma. Improving knowledge and attitudes may encourage better practices among parents of children with asthma.

## Background

Asthma is one of the common chronic diseases among children. Increases in asthma prevalence, morbidity and mortality have intensified public health concern [1]. In 2000, asthma affected an estimated 0.12–3.34% of children (aged 0–15) in China, with a wide range of associated problems and high health costs [2]. School absenteeism of more than 10 days per year occurs among 42.47% of children with asthma. It is the leading cause of ambulatory visits and hospitalizations among children [3]. This potentially fatal chronic disease carries a significant burden for patients, families and communities [4].

Asthma management refers to the monitoring and control of symptoms and the prevention of exacerbations. It aims to improve long-term control and reduce the need for emergency treatment [5]. However, many children with either diagnosed or undiagnosed asthma go untreated, resulting in a poor quality of life [6]. A wide gap exists between recommended and actual practice, owing to educational barriers and a lack of adequate asthma-related knowledge [7]. According to childhood asthma guidelines, parent training to improve their asthma-related knowledge, altitudes and practices (KAP) should be included in routine clinical care [1,8]. Asthma-related knowledge includes an understanding of pathological mechanisms, medications and prevention [9]. A good attitude is reflected by confidence and positive relationships with physicians [10]. Good practices refer to timely intervention and active management with appropriate care [11].

To improve childhood asthma management, an assessment of parent KAP is a significant requirement. The primary objective of this study was to document the KAP of parents of children with asthma and identify how knowledge and attitudes relate to practices. Our secondary objective was to identify the factors associated with parent KAP scores. A cross-sectional survey of KAP was conducted on parents of children with asthma in China’s 29 provinces and cities (except for Xinjiang and Tibet). Parent KAP were measured by the three domains of an Asthma Knowledge, Attitudes and Practices Questionnaire. Survey findings indicate that asthma-related education for parents/guardians is required to improve asthma care and management.

## Methods

### Design and ethics

Using a questionnaire, we conducted a cross-sectional survey of the KAP of parents of children with asthma. We constructed the study questionnaire based on the methodology of KAP studies conducted in other countries, and adapted it to the Chinese situation and culture.

The Medical Ethics Committee of the Capital Institute of Pediatrics gave approval for the study and each respondent provided written informed consent.

### Participants

The study was conducted in the asthma clinics of tertiary hospitals in 29 cities (capital cities of provinces, municipalities and autonomous regions). By simple random sampling, we selected 2960 children with asthma aged 0–14 years (born between 1 January 1995 and 31 October 2009) who presented at a hospital between 1 May and 31 October, 2009, and had been diagnosed with asthma for at least 3 months. The diagnoses must have conformed with the guidelines for the diagnosis and management of childhood asthma of the Chinese Medical Association Pediatric Respiratory Study Group.

### Questionnaire

The KAP questionnaire was designed by asthma experts by referring to the Asthma Insights and Reality in Europe [12,13] survey and the Asthma Control Test [14]. After pilot testing, the questionnaire was discussed and revised by the asthma experts until the final version was formed. The 50-item questionnaire comprised five parts: (1) general information about the parents of children with asthma; (2) child conditions and asthma control in the past 12 months, (3) parent knowledge; (4) parent attitudes and beliefs, and (5) parent practices. The questionnaires were scored on 30 items regarding parent asthma-related KAP, with one point for every correct response. Incomplete questionnaires were not scored. All questionnaires were completed by investigators through face-to-face interviews with children with asthma and their parents to ensure validity.

### Analysis of data

All data were entered into EpiInfo version 6.0 (CDC, Atlanta, USA) in duplicate. Data were categorized and analyzed using Statistical Package for Social Sciences version 13.0 (SPSS Inc., Chicago, IL). Quantitative variables were described by means and standard deviations, and qualitative variables by frequency distributions, proportions and percentages. Logistic regression and chi-squared tests were used to determine the associations between factors. A *p*-value < 0.05 was considered statistically significant.

## Results

### Patient (child) characteristics

In total, 2485 valid questionnaires were collected, a response rate of 83.95%. There were 1660 (66.8%) male children and 825 (33.2%) female children. Their average age was 7.20 ± 3.09 years, and the average disease course was 2.81 ± 2.05 years. More than a third (37.6%) of the children had a history of food allergy, nearly half (48.5%) had a history of eczema, more than half (58.59%) had rhinitis and nearly a third (29.7%) had a family history of asthma.

### Parent knowledge, attitudes and practices

In the 2485 valid questionnaires, the average combined KAP score was 17.11± 3.92, with level expression: the fourth level (< 18 points) 51.43% (1278/2485), the third level (18–20 points) 29.58% (735/2485), the second level (21–24 points) 16.86% (419/2485), and the highest level (> 24 points) 2.13% (53/2485).

### Parent asthma-related knowledge

The knowledge component of the questionnaire contained 13 items. The average score was 5.69 ± 2.10 and 18.31% of parents scored ≥ 8. Many parents (63.98%) knew that asthma is a chronic allergen-induced airway inflammatory disease. Only 6.08% (151/2485) knew that “wheezing > 3 times, coughing > 4 weeks, > 6 respiratory infections in the last year and relief after using bronchodilator” could suggest asthma. Almost two thirds (63.58%) of parents knew that wheezing more than three times suggests asthma. By contrast, only 23.74% (590/2485) recognized that a chronic cough may indicate asthma. “Self-withdrawal, catching a cold, exposure to allergens, strong emotional changes and cold stimulation” were identified by 11.03% (274/2485) of parents as precipitants of asthma. “Repeated strenuous coughing, chest tightness with restricted breathing and dry cough after exercise or sleep” were identified by 20.97% (521/2485) of parents as suggestive of an asthma attack. Parents exhibited a low level of asthma-related knowledge, with a better understanding of the nature of asthma, but a lack of awareness of clinical manifestations of the disease and the indicators of acute attacks.

### Parent attitudes and beliefs

The attitudes component of the questionnaire contained seven items. The average score was 5.23 ± 1.283 and most parents (89.58%) scored ≥ 4. A total of 83.30% (2070/2485) of parents knew that asthma is controllable with regular medication. Most (84.67%) believed that their child could participate in sports if their asthma was under control, and of these parents, 60.31% (1269/2104) believed that children with asthma could exercise as much as healthy children. However, 33.60% (835/2485) of parents would allow their children to participate in minor sports only. In items related to adherence with inhaled corticosteroids, 67.32% (1673/2485) of parents worried about negative effects on children’s growth, 40.56% (1008/2485) worried about drug dependence, and 23.98% (596/2485) were worried about potential harm to their child’s intelligence. Regarding access to asthma knowledge, 80.63% (2003/2485) of parents preferred to communicate with physicians.

### Parent practices

The practice component of the questionnaire contained 10 items. The average score was 6.19 ± 1.57, and more than half of the parents (67.89%) scored ≥ 6. Regarding examination, two-thirds (68.25%) of children had undergone allergen tests, of which 76.24% (1293/1696) had undergone skin prick tests and 66.96% (1664/2485) had undergone pulmonary function tests. However, only 25.03% (622/2485) had used a peak flow meter to monitor their daily condition, and only 7.00% (174/2485) had used the children’s Asthma Control Test questionnaire. Regarding monitoring, 73.88% (1836/2485) of parents of children with well-controlled asthma regularly took their child to see a physician, among which 67.05% (1231/1836) visited every 1–3 months. Most parents avoid their child being exposed to tobacco smoke (94.78%) and plush toys (88.17%). Many (81.53%) parents of children with controlled asthma insisted on their child adhering to their medication regimen, of which 78.33% (1587/2026) adhered to the correct use of inhaled corticosteroids/compounds and 31.44% (637/2026) to oral leukotriene receptor modulators. In 1456 children with comorbid allergic rhinitis, 34.68% (505/1456) adhered to the correct use of nasal steroids. In addition, 18.87% (469/2485) of parents also used antibiotics as supplementary therapy, regardless of whether their child exhibited symptoms associated with infection.

### Influence of parent knowledge and attitudes on practices

Table 1 shows that parent asthma-related knowledge (dichotomized by cut-off score of 8) was associated with eight aspects of pulmonary function testing, regular physician visits, asthma monitoring with a peak flow meter and the C-CAT questionnaire, avoidance of asthma triggers (including smoke and plush toys), using an inhaled β2 receptor agonist and adherence to medication regimen. There were no associations between parent knowledge and allergen testing or using antibiotics.

**Table 1:** Influence of parent asthma-related knowledge on practices

	**χ2**	**Sig.**	**OR**	**95% CI**	
				**lower**	**upper**
Review of lung function	9.103	0.003	1.414	1.128	1.771
Regular physician visit	19.612	0.000	1.780	1.376	2.034
Monitor with a peak flow meter	2.950	0.000	5.921	4.767	7.353
Monitor with the C-CAT	1.801	0.000	7.034	5.108	9.688
Avoid passive smoking	17.272	0.000	4.882	2.138	11.148
Avoid plush toys	29.676	0.000	3.404	2.136	5.424
Acute inhaled of β2 receptor agonist	36.448	0.000	1.89	1.534	2.328
Adherence to medication regimen	46.803	0.000	3.406	2.353	4.932

Table 2 shows that parent asthma-related attitudes (dichotomized by a cut-off score of 4) were significantly associated with allergen testing, pulmonary function testing, regular physician visits, monitoring with a peak flow meter and the C-CAT questionnaire, smoke avoidance, using an inhaled β2 receptor agonist and better adherence to medication regimen. Better practices were associated with positive parent attitudes.

**Table 2:** Influence of parent asthma-related attitudes on practices

	**χ2**	**Sig.**	**OR**	**95% CI**	
				**lower**	**upper**
Allergen testing	5.175	0.023	1.362	1.043	1.778
Review of lung function	5.920	0.015	1.387	1.065	10806
Regular return visit	55.834	0.000	2.582	2.000	3.333
Monitor with a peak flow meter	23.396	0.000	2.470	1.694	3.601
Monitor with the C-CAT	6.799	0.009	2.528	1.229	5.202
Avoid passive smoking	7.765	0.005	1.930	1.206	3.088
Acute inhaled of β2 receptor agonist	21.358	0.000	2.113	1.529	2.919
Adherence to medication regimen	3.862	0.000	11.300	8.533	14.964

### Factors associated with parent KAP questionnaire scores

A logistic regression analysis was used to determine the effects of multiple factors on parent KAP score. The dependent variable was KAP questionnaire score (dichotomized by cut-off score of 18). The independent variables are parent education, monthly family income, course of asthma in children, food allergy history, eczema history, coexist with allergic rhinitis, family history of asthma, regular return visits for asthma control, frequency of review. Table 3 shows that food allergy, rhinitis, regular physician visits, frequency of physician visits and parent education had positive associations with KAP scores.

**Table 3:** Factors associated with parent KAP scores

	**B**	**S.E**	**Wald**	**df**	**Sig.**	**Exp(B)**	**95.0%C.I.**	
							**lower**	**upper**
Parent education	0.386	0.058	43.893	1	0.000	1.471	1.312	1.649
Food allergy history	0.227	0.102	4.930	1	0.026	1.255	1.027	1.533
Allergic Rhinitis	0.592	0.101	34.127	1	0.000	1.808	1.482	2.206
Regular return visits	0.910	0.287	10.050	1	0.002	2.485	1.415	4.362
Frequency of review	0.155	0.073	4.461	1	0.035	1.167	1.011	1.347
Constant	−3.362	0.296	128.817	1	0.000	0.035		

## Discussion

The Global Initiative for Asthma (GINA) [1] and the asthma guidelines for prevention and treatment written by an expert panel from the National Heart, Lung, and Blood Institute emphasize the importance of promoting a standardized classification of asthma treatment [15]. In this analysis of the KAP of Chinese parents of children with asthma, a wide gap was observed between recommended and actual practices, and their overall asthma-related knowledge was insufficient. Furthermore, asthma-related knowledge was associated with deficiencies in the care process. Therefore, we suggest that further investigation is required to develop asthma-related education programs for parents.

Rea *et al*. [16] found that a lack of asthma-related knowledge and improper management of non-compliance are risk factors for asthma fatality. We found a direct association between parent knowledge and asthma prevention and management practices. Therefore, we suggest that the most important component of childhood asthma treatment is parent education to improve their asthma management practices.

KAP education programs for parents of children with asthma are well-established in some Western European countries [17]. The community system in China is in a developing stage, and is far from perfect. Examining the KAP of parents of children with asthma and developing asthma-related education and management programs have not been given sufficient attention. This is the first study of parent asthma-related KAP and their determinants in China.

### Parent asthma-related knowledge

In this study, more than half (51.34%) of the parents scored ≤ 18 in the KAP questionnaire, indicating low KAP among most parents. Similar to the results of a 2009 asthma-related KAP survey in Beijing, two-thirds (63.98%) of the parents in our study knew the nature of asthma [18]. Conversely, in a similar survey in India, the majority of respondents (54%) were not aware of the nature of asthma [19]. Nevertheless, only 18.31% of parents in our study correctly answered ≥ 60% of the questions.

Studies have shown that asthma, especially cough variant asthma (CVA), is the leading cause of chronic cough in children [20,21]. CVA is defined by GINA as a special type of asthma with cough as the sole or main symptom that is more common in children [1,22]. In Chinese guidelines, CVA is diagnosed as an isolated persistent cough, with increased airway responsiveness or abnormal expiratory flow rate variability that is effectively treated with bronchodilators [23]. In this study, only one fourth (23.74%) recognized that a chronic cough may indicate asthma. Children with persistent cough over more than four weeks should be considered for a diagnosis of chronic cough. Parent asthma-related knowledge is at a low level in China, with a lack of awareness of asthma clinical manifestations or the triggers to acute attacks. As measured by the Knowledge, Attitude, and Self-Efficacy Asthma Questionnaire in the United States, patients with asthma had limited asthma-related knowledge, with only half of the patients correctly answering ≥ 50% of the questions [24].

Childhood asthma management requires multiple complex tasks. Parents need to understand the diverse triggers and basic mechanisms of an asthma attack, and to understand the necessity of maintenance medication. Parents also need to learn to monitor lung function, use C-CAT, dose rescue medications, recognize exacerbations early and adopt emergency care [25]. In one study, 115 parents of children in Head Start Centers received asthma-related education. The results showed that the provision of education improved asthma and healthy home-knowledge, and that 98.4% of the parents made changes in their households [26].

### Parent attitudes

In our survey, about 33.60% (835/2485) of parents would allow their children to participate in minor sports, even if their asthma was under control. This indicates that there is doubt among parents about whether children with asthma can participate in sport. In GINA, it is proposed that an indicator of asthma control is being able to participate in as much sport as healthy children [1]. One study showed that children adhering to their asthma medication can participate in sport, improve their physical fitness, reduce the frequency of asthma attacks and prevent further exacerbation [27].

Among the children who were not adhering to their medication regimen, 67.32% (1673/2485) of parents worried about negative effects on children’s growth, and 23.98% (596/2485) were worried about potential harm to their child’s intelligence. This indicates that the parents’ strongest fears regarding medication are related to side effects. Despite this, most research shows that low-dose inhaled corticosteroids do not cause growth retardation, abnormal bone calcium/phosphorus metabolism or other systemic side effects [28,29].

Regardless of the actual or desired pathways that parents obtain asthma-related knowledge, communication with medical staff is given top priority. From the perspective of many parents, good communicative relationships with health professionals are vital for encouraging progress through childhood asthma management stages [30]. A United States study showed that parents who receive a written action plan in the pediatric emergency department are more confident in their ability to provide care for their child during an asthma exacerbation [31]. We should support the efforts of professionals to disseminate information to improve asthma knowledge.

### Parent practices

Guidelines recommend that the assessment of asthma severity and control should be carried out early and on a regular basis. Compared with a 1999 survey of the asthma situation and trends in Europe [12], this study found that the situation in China is better in terms of children taking pulmonary function tests and poorer in terms of regular monitoring. Given the low rates of peak flow meter and C-CAT test use, parents should be encouraged to use both self-monitoring tools to better monitor their child’s asthma status and provide timely and effective treatment.

Previous respiratory research has identified that children with allergic rhinitis in infancy have twice the risk of being physician-diagnosed with asthma at age 11 years [32]. Future education programs should emphasize that upper and lower respiratory tract allergic diseases should be treated together, and combined therapy should be used for allergic rhinitis and asthma [33]. In recent years, antibiotic abuse in the treatment of children with asthma has become increasingly prevalent. Parents of children with asthma need to be informed that antibiotics should only be taken for symptoms associated with infection and with the support of laboratory indications.

This study provides suggestions for improving the quality of parent/guardian care of children with asthma. In primary care, detailed and accurate allergen identification is essential for the proper diagnosis of allergic asthma and its successful treatment. Parent education and practical advice about allergen avoidance should be provided. Finally, referral for more extensive investigation and management should be considered [34].

This study was carried out in 29 cities (capital cities of provinces and municipalities and autonomous regions, with different economic levels and geographical environments). The sample size is relatively large and the results are representative of urban China. However, they may only be generalizable to cities, not rural areas. A further limitation is that many factors analyzed in this study were based on responses to questionnaires; therefore, recall bias may have been present.

It is possible that asthma control status affects parent asthma-related KAP. Further research is required to determine if interventions would be beneficial.

## Conclusions

In this study, consistencies between parent knowledge, attitudes and practices were identified. This indicates that improved asthma knowledge and attitudes can encourage parents to correctly monitor their child’s asthma condition and better manage and adhere to their medication regimen. In this survey, parent education had a positive association with KAP scores. Education level will affect the parents’ ability to acquire knowledge. Higher-educated parents have more asthma knowledge and can better provide the appropriate care. A history of food allergy and rhinitis was positively associated with parent KAP scores. This indicates that these parents are more likely to seriously consider the allergy status of their children. Regular physician visits, when asthma is controlled, can bring more opportunities for asthma education to parents.

## Competing interests

The authors declare that they have no competing interests.

## Authors’ contributions

JZ conceived of the study idea, developed the design and edited the manuscript. MX participated in study development and edited the manuscript. GQZ participated in the study design, performed the statistical analysis and helped draft the manuscript. QQH, QYC and JB participated in the study development and data entry. All authors read and approved the final manuscript.

## Pre-publication history

The pre-publication history for this paper can be accessed here:

http://www.biomedcentral.com/1471-2431/13/20/prepub
